# Quality of Life in Cutaneous T-cell Lymphoma Patients Receiving Mogamulizumab: Important Factors to Consider

**DOI:** 10.3390/cancers15010032

**Published:** 2022-12-21

**Authors:** Rosanne Ottevanger, Sylvia van Beugen, Andrea W. M. Evers, Rein Willemze, Maarten H. Vermeer, Koen D. Quint

**Affiliations:** 1 Department of Dermatology, Leiden University Medical Center, Albinusdreef 2, 2333 ZA Leiden, The Netherlands; 2 Health, Medical and Neuropsychology Unit, Institute of Psychology, Leiden University, 2333 AK Leiden, The Netherlands

**Keywords:** cutaneous T-cell lymphoma, quality of life, mogamulizumab, mycosis fungoides, Sézary syndrome, treatment expectations, treatment satisfaction, erythroderma

## Abstract

**Simple Summary:**

Patients with erythrodermic cutaneous T-cell lymphoma often experience severe symptoms. This study provides an insight into the impact of the disease, mogamulizumab treatment, and essential patient-reported outcomes. The data on patients’ expectation, quality of life, and treatment satisfaction can especially be used in the counseling of future patients.

**Abstract:**

Background: Erythrodermic cutaneous T-cell lymphoma (E-CTCL) is associated with a poor prognosis and severe symptoms. Objective: To establish insights into the quality of life (QoL), expectations, and treatment satisfaction of E-CTCL patients receiving mogamulizumab. Methods: Outcomes of this prospective cohort study conducted between September 2020 and August 2021 at the Leiden University Medical Center included the dermatology-specific QoL (Skindex-29), health-related QoL (RAND-12), degree of itch, pain, and fatigue (Visual Analogue Scale), patient’s expectations, and treatment satisfaction (Client Satisfaction Questionnaire-8 (CSQ-8)), measured at baseline and after six months. Results: 13 patients with E-CTCL were included. Most patients anticipated a positive treatment effect on symptoms. Five patients (46%) improved one or more clinical categories regarding the symptoms domain, six (55%) regarding emotions, four (36%) regarding functioning, and four (36%) regarding the overall Skindex-29 score compared to baseline. The Mental Component Score clinically improved from 31 (IQR 29–51) at baseline to 38 (IQR 25–51). The median VAS itch improved significantly from baseline (8 (IQR 7–10) vs. 3 (IQR 1–8), *p* = 0.024). Most patients (n = 7) were “very satisfied” with their treatment. Limitations: There was a limited number of patients due to the rarity of the disease. Conclusion: In general, mogamulizumab has a favorable effect on biochemical- and dermatology-specific QoL and physical functioning in some patients, with high treatment satisfaction. Itch especially improved over time in most patients. The treatment satisfaction was generally high. Mogamulizumab seems to be an effective treatment that improves the QoL in patients with E-CTCL.

## 1. Introduction

Primary cutaneous T-cell lymphomas (CTCL) represent a heterogeneous group of non-Hodgkin lymphomas presenting in the skin without evidence of extracutaneous disease at the time of diagnosis [1]. Mycosis fungoides (MF) and Sézary syndrome (SS) are considered as the classic types of CTCL [2]. Generally, MF has an indolent disease course with slow disease progression over years or decades from patches and plaques to eventually tumors and/or erythroderma, and in some cases extracutaneous disease. SS is a rare leukemic variant of CTCL, marked by the triad of erythroderma, generalized lymphadenopathy, and blood involvement [1]. The incidence of SS in The Netherlands was 0.075 per 100,000 persons in 2019 and has been rising in the last two decades [3].

Erythrodermic CTCL (E-CTCL) is a classic CTCL (MF or SS) in which patients present with a complete red skin [1,4]. E-CTCL is generally associated with a poor prognosis and severe symptoms (such as itch and scaling) with a consequent negative effect on quality of life (QoL) [1,2,4,5,6].

Due to the chronic symptomatic course and the scarce curative treatment options of CTCL, lifelong palliative therapy is necessary for suppressing E-CTCL symptoms, making QoL measurements a necessary tool for validating therapy besides clinical observations [5,7]. In addition, there are also limited treatment options for E-CTCL patients, e.g., systemic treatment such as mono- or poly-chemotherapy. Positive effects are often short-lasting and adverse effects are often seen. In 2018 the CCR4 antibody mogamulizumab was approved in the European Union by the EMA for the treatment of refractory or relapsed CTCL [8,9,10]. 

Currently, only a few studies have addressed the possible improvement of dermatology-specific and general QoL in patients treated with mogamulizumab [7,9,11]. In addition, due to the chronic aspect and general incurability of the disease, it is important to gain insight into the current expectations of the treatment and treatment satisfaction of patients receiving mogamulizumab and their relationship with QoL. Expectations of the treatment are particularly relevant as possible predictors of treatment success, as frequently reported in the placebo literature [12]. Awareness of the specific expectations and affected domains of QoL in patients with CTCL could help to improve counseling and expectation management.

This prospective study aimed to establish patient-reported outcomes to provide insights into clinical practice and understanding of patients’ expectations, QoL, and treatment satisfaction in E-CTCL patients receiving mogamulizumab that can be used for counseling and expectation management.

## 2. Materials and Methods

For this prospective cohort study, patients with E-CTCL receiving mogamulizumab were recruited between 1 September 2020 and 31 August 2021 from the outpatient clinic of the Dermatology department in the Leiden University Medical Center (LUMC). The LUMC is the national referral center for CTCL in The Netherlands. In all cases, the diagnosis was based on the clinicopathologic criteria of the WHO-EORTC classification and confirmed by an expert panel of dermatologists and pathologists during one of the periodical meetings of the Dutch Cutaneous Lymphoma Working Group. 

Patients were invited to participate if they were aged over 18 years, were starting treatment with mogamulizumab, and were able to provide formal written consent. Patients were excluded if they were unable to speak the Dutch language.

Within the study period 14 patients were eligible for participation of which one patient declined participation. E-CTCL was regarded as CTCL patients with erythroderma independent of the blood and lymph node involvement, including patients with SS (stage IV), MF (stage III–IV), and folliculotropic MF (stage III–IV). At the time of diagnosis all patients met the criteria of MF or SS determined by the World Health Organization-European Organization for Research and Treatment classification [1]. The group of E-CTCL patients is a very small proportion of patients with CTCL and not every patient with E-CTCL was receiving mogamulizumab at the time of this study. Most patients with E-CTCL who received mogamulizumab are treated at the national referral center. This study was approved by the institutional medical ethical review board (N20.052). 

At the start of the mogamulizumab, patients were asked to complete several questionnaires related to their generic- and dermatology-specific QoL and their expectations of the treatment. After six months, QoL was re-evaluated, and an evaluation of treatment satisfaction was performed. In addition, baseline characteristics (age, sex, comorbidities, age at diagnosis, and time since diagnosis) were extracted from their medical records. The medical history was used to calculate the classic Charlson Comorbidity Index (CCI) without considering the primary disease and was categorized into low (CCI 0–1) and high numbers of comorbidities (CCI ≥ 2) [13]. The disease was evaluated based on progression or regression of lymph nodes, blood involvement, and/or skin involvement. The lymph node and skin involvement were measured based on the radiology report of the Positron Emission Tomography-Computed Tomography scan (PET-CT). Evaluation of blood involvement was measured with the CD4/CD8 ratio. 

### 2.1. Outcome Measurements

The primary outcome was the dermatology-specific QoL measured by the Skindex-29. Secondary outcomes were generic health-related QoL as measured by the RAND-12, treatment expectations, and treatment satisfaction as measured by the Client Satisfaction Questionnaire (CSQ) [14,15,16,17]. In addition the CD4/CD8 ratio, lymphadenopathy and visceral involvement on PET-CT imaging were evaluated to give insight into the clinical response. 

### 2.2. Expectations Questionnaire

To assess treatment expectations, a 6-item questionnaire was developed for the purpose of this study, since expectations have to be tailored to the specific treatment outcome. The expectations questionnaire was only assessed at the start of the study. Items were related to the expected effect of the treatment on pain, time consumption, mood, energy, the anticipated treatment effect, and adverse events. The Cronbach’s alpha was found to be 0.56 and was calculated with all six items of the questionnaire. 

### 2.3. Skindex-29

The Skindex-29 is a validated 29-item questionnaire that specifically addresses the QoL in patients with skin disease with good internal consistency (Cronbach’s alpha = 0.76–0.86) [18]. Individual items measure the frequency of affected QoL on 5 levels using 5-point scales ranging from 1 (‘never’) to 5 (‘all the time’). The individual items can be categorized in 3 separate domains: symptoms, emotions, functional limitations, and as a total score. Each of the domains have pre-defined cut-off points. These are ≥39, ≥42, and ≥52 for mild, moderately, and severely impaired, respectively, for the symptoms domain; ≥24, ≥35, and ≥39, respectively, for the emotions domain; and ≥21, ≥32, and ≥37, respectively, for the functional limitation domain. The cut-off points for the total Skindex-29 score are ≥25 for mild, ≥32 for moderate, and ≥44 for severe impairment. Scores below the lowest cut-off points for mildly impaired were regarded as unaffected [17]. Skindex-29 was measured at the first and second point of time. 

### 2.4. RAND-12

The RAND-12 Health Survey (RAND-12) measures the impact of disease with a physical component score (PCS) and mental component score (MCS). The domains range from 0 to 100, with higher scores correlating with a better health status. A score difference of 3 to 5 points is considered clinically meaningful [15]. The RAND-12 was measured at baseline and at follow-up.

### 2.5. Itch, Pain, and Fatigue

The degree of itch, pain, and fatigue in the last 4 weeks was reported with a visual analogue score (VAS) (0–10) for each item at baseline and follow-up [16]. 

### 2.6. Client Satisfaction Questionnaire (CSQ)

The CSQ was used to establish treatment satisfaction [19]. The questionnaire consists of 8 items. The CSQ was conducted 6 months after the start of mogamulizumab. The total score ranges from 25–100 with higher scores indicating higher satisfaction. No clinical correlated categories can be derived from the total score. 

The study was reported following the Strengthening the Reporting of Observational studies in Epidemiology (STROBE) guidelines [20].

### 2.7. Statistical Analysis

Statistical analysis was performed with IBM SPSS statistics 25, Armonk, NY, USA. Sum scores for all items were calculated according to the corresponding instrument manuals. Continuous data were reported as median and interquartile range (IQR) due to non-normal distribution and categorical data as number and percentage (%). Normality of data distribution was tested with the Kolmogorov–Smirnov test. The Wilcoxon rank test was performed to establish potential statistical change over time. Statistical tests were performed for the total group of patients, including the non-affected category in the case of Skindex-29. The correlations of the expected treatment effect and treatment satisfaction (CSQ) with Skindex-29 scores at baseline and after six months were assessed using the Pearson R correlation analysis. A coefficient of ≤0.19 was considered very weak, 0.20–0.39 weak, 0.40–0.59 moderate, 0.60–0.80 strong, and 0.80–1 very strong.

## 3. Results

### 3.1. Study Population

In total, 13 patients with E-CTCL were included with a median age of 68 years (Table 1). Most patients were diagnosed with SS (77% (n = 10/13)). Two patients died prior to the end of follow-up. For the CD4/CD8 ratio and PET-CT findings over time see Table 2. The CD4/CD8 ratio was positively influenced or stable in all patients. Three patients (25%) showed progression of disease on the PET-CT (Table 2).

### 3.2. Patient Expectations

Patients generally expected to experience little (“somewhat” (n = 5/12)) to no (“not at all” (n = 7/12)) pain during the treatment (Table 3). Most patients (n = 7/12) “strongly” expected the treatment and recovery time to be time-consuming. Patients “not at all” (9/12) to “somewhat“ (n = 3/12) expected the treatment to affect their mood. Most patients “not at all” (n = 4/12) to “somewhat” (n = 8/12) expected to feel less energized during and after the treatment. Most patients anticipated a positive treatment effect of the mogamulizumab symptoms. Three out of eleven patients “somewhat”, four out of eleven “strongly”, and four out of eleven patients “totally” expected a positive effect on their treatment. Only one patient “not at all” expected adverse events from the treatment, while eight out of eleven “somewhat” and two “strongly” expected adverse events.

### 3.3. Skindex-29

At baseline eight out of thirteen patients (62%) were severely affected regarding the total Skindex-29 score (Table 4). Statistically the scores for the symptoms (*p* = 0.14), emotions (*p* = 0.54), functioning (0.689), and overall domain (0.610) did not differ at baseline and after 6 months of therapy. However, five patients (46%) did improve on at least one or more clinical categories regarding the symptoms domain, six (55%) regarding emotions, four (36%) regarding functioning, and four (36%) regarding the overall score. 

### 3.4. RAND-12

The median PCS at the start of the study period was 35 (IQR 32–44) and 32 (IQR 30–45) at follow-up (*p* = 0.59) (Table 4). The PCS clinically meaningfully improved in five (46%) of the patients. The MCS was 31 (IQR 29–51) at baseline and 38 (IQR 25–51) at follow-up, which is a clinically meaningful change, although not statistically significantly different (*p* = 0.86). The MCS clinically meaningfully improved in six patients (55%). 

### 3.5. Itch, Pain, and Fatigue

The intensity of experienced itch, pain, and fatigue was high at baseline (VAS 8 (7–10), 7 (2–8), and 6 (1–8) respectively) (Table 4). In total eight patients (73%) noticed improvement in itch based on their provided VAS scores, six (55%) noticed improvement in pain, and six (55%) noticed some improvement in fatigue. Overall, the median VAS score for itch improved significantly (*p* = 0.02) (Table 1). Pain and fatigue did not statistically improve (*p* = 0.12 and *p* = 0.72, respectively).

### 3.6. Client Satisfaction Questionnaire (CSQ)

Overall, satisfaction with mogamulizumab treatment was high (Table 5). The median CSQ score was 81 (IQR 59–79). Out of the 11 surviving patients, seven (64%) were “very satisfied” overall, only two patients (18%) were “indifferent or mildly dissatisfied”, and two (18%) were “mostly satisfied” with the overall treatment. 

### 3.7. Correlations with Patients’ Expectations, QoL, and Treatment Satisfaction

The anticipated treatment effect was strongly correlated with the baseline total score of the Skindex-29 (R 0.78, *p* = 0.005), meaning that patients with a lower dermatology-related QoL had higher expectations of the mogamulizumab therapy. In addition, a lower dermatology-specific QoL (higher total Skindex-29 score) after 6 months was also strongly associated with a lower treatment satisfaction as measured by the CSQ (R 0.79, *p* = 0.004). There was no significant correlation between the anticipated treatment effects and the total Skindex-29 score after 6 months (R 0.23, *p* = 0.52). Also, the total baseline Skindex-29 was not significantly correlated with the total Skindex-29 score after six months (R 0.73, *p* = 0.73). 

## 4. Discussion

This study shows that E-CTCL has a profound effect on the QoL. Patients generally expected the mogamulizumab to have a positive effect. However, they strongly expected the treatment and recovery time to be time-consuming and did anticipate adverse events. The mogamulizumab infusion, in general, has a favorable effect on biochemical and some clinical parameters. The dermatology-specific QoL, physical functioning, and especially itch improved over time. None of the surviving patients experienced a worsening of their QoL. The treatment satisfaction of patients receiving the mogamulizumab therapy was generally high. The anticipated treatment effect was strongly correlated with the baseline dermatology-related QoL. Also, a lower dermatology-specific QoL after six months was strongly associated with a lower treatment satisfaction. 

While patients with E-CTCL have a poor prognosis, mogamulizumab is a relatively novel therapy for CTCL patients and has shown to have a positive effect on progression-free survival (median 8 months (95% CI (6–10)) [9]. In addition, it has shown an overall skin response in approximately 30–37% of patients [9,21]. However, as debilitating symptoms have been described, the clinical effects on dermatology-specific and general QoL are also very important. In this study 15% (two out of 13) died during the study period (one disease-related and one not related). 

In general, this study has shown benefits in approximately half of patients regarding dermatology-specific and general functioning. A previous longitudinal analysis also found a benefit of mogamulizumab therapy on the symptoms, emotional, and functioning scales of the Skindex-29 [7]. 

Patients with the greatest symptom burden and functional impairment showed the most QoL benefit from the therapy [7]. This was also found in some, but certainly not in all patients, as some patients in the severely affected QoL categories remained severely affected. However, a decreased risk of experiencing a more rapid deterioration in QoL has been found previously [7].

The results of this study confirm that expectations and treatment satisfaction are related to dermatology-specific QoL. Patients’ expectations and treatment satisfaction for mogamulizumab have, to our knowledge, not been described in the literature before. Counseling about the treatment effects and potential adverse events are very important, especially in patients with severe symptoms and a poor life expectancy. It has been proven that treating physicians can induce positive and negative expectations in patients by expressing their view on the potential outcomes and medication’s efficacy. It is therefore important to adequately address patient expectations with realistic outcomes. Especially, as most patients in our study expected strong effects of the anti-CCR4 therapy [22,23].

It should be realized that E-CTCL has a serious effect on health status and QoL of patients. As a proportion of patients did not experience an improvement regarding (dermatology-specific) QoL, clinicians should be aware that, due to the lack of other therapeutic options, these patients potentially also need additional supportive care of, e.g., medical psychologists. 

Patients in our study expected to experience some adverse events from the anti-CCR4 therapy. A previous review on safety and efficacy found that mogamulizumab had an acceptable toxicity and that the most common all-grade adverse events were lymphopenia, infusion reaction, fever, rash, and chills [24]. Grade 3–4 adverse events are reported in up to 41% of patients [9].

This study has some limitation due to the small sample size. However, in the context of this very rare disease it is still a sizable cohort. Although this was a monocenter study, it was performed in the national referral center for cutaneous lymphomas in The Netherlands. No clinical response regarding mSWAT was performed as no clinical visits were scheduled for research purposes. 

Despite some limitations, this study provides a relatively unique insight on the impact of the disease, treatment, and essential patient-reported outcomes. The data on the patients’ expectations and treatment satisfaction can especially be used in the counseling of future patients. 

## 5. Conclusions

Patients with erythrodermic CTCL experience a profound effect on the QoL. Mogamulizumab seems to be an effective treatment in improving the QoL of patients with E-CTCL. In general, mogamulizumab has a favorable effect on biochemical- and dermatology-specific QoL and physical functioning in some patients, with high treatment satisfaction. None of the patients experienced a worsening of their QoL during the mogamulizumab therapy. The most beneficial effect of mogamulizumab was on itch. The anticipated treatment effect and treatment satisfaction were strongly correlated to the dermatology-specific QoL. These data can be used for counseling and expectation management of patients with E-CTCL and their caregivers.

## Figures and Tables

**Table 1:** Patient characteristics.

Patient Characteristics Patient Nr.	Age (Years)	Sex	Age at Diagnosis (Years)	Time Since Diagnosis (Years)	CCI	Disease Stage
*Patient 1*	75	F	66	9	3	FMF III
*Patient 2*	48	M	41	7	1	MF IV
*Patient 3*	79	M	73	6	4	SS
*Patient 4*	72	M	65	6	0	SS
*Patient 5*	83	F	82	0	3	SS
*Patient 6*	64	F	59	4	0	SS
Patient 7 *	62	F	60	1	1	SS
*Patient 8*	72	M	69	2	1	SS
*Patient 9*	53	F	52	0	0	SS
Patient 10 *	68	F	66	1	4	SS
*Patient 11*	70	M	68	1	1	MF III
*Patient 12*	42	F	60	1	0	SS
*Patient 13*	60	M	59	1	2	SS
Total sample (*n* = 13)	68 (61–74)	7 (55%) F	65 (59–69)	1 (1–6)	1 (0–3)	-

Numbers are displayed as number (%) or median (interquartile range). CCI, Charlson Comorbidity Index; F, female; M, male; MF, Mycosis fungoides; FMF, Folliculotropic Mycosis fungoides; SS, Sézary Syndrome. * Died during follow-up.

**Table 2:** Lab and imaging characteristics at inclusion and end of the study period.

	Inclusion						End of Study					
Patient Nr.	Lab			PET-CT			Lab			PET-CT		
	CD4/8 Ratio ∞	CD4 10^6^/L	CD8 10^6^/L	C	VI	LN	CD4/8 Ratio *	CD4 10^6^/L	CD8 10^6^/L	C	VI	LN
*Patient 1*	9.71	4954	4465	+	−	R	1.22	323	265	−	−	−
*Patient 2*	26.92	4054	151	+	−	−/R	1.06	148	140	++	−	+
*Patient 3*	19.37	1281	66	+	−	−	1.18	306	259	−	−	−
*Patient 4*	37.29	283	8	−	−	+	7.89	250	32	−	−	−/R
*Patient 5*	2.59	354	137	−	−	+	9.41	4056	431	+	−	+
*Patient 6*	88.4	7480	85	−	−	+	1.32	204	147	−	−	−
*Patient 7*	5.17	1003	194	+	+	+	0.27	193	701	++	+	++
*Patient 8*	3.04	362	119	−	−	−	0.43	53	123	−	−	+
*Patient 9*	2.19	116	76	+	+	−	0.74	133	180	+	−	+
*Patient 10*	5.45	1847	338	−	−	−/R	6.62	1011	153	−	−	−/R
*Patient 11*	1.53	2848	1865	+	−	+	0.26	447	1713	++	+	++
*Patient 12*	10.95	704	64	−	−	−/R	12.43	2477	199	−	−	−/R
*Patient 13*	6.29	401	64	−	−	−/R	6.07	936	154	−	−	+

PET-CT, Positron Emission Tomography-Computed Tomography scan; C, Cutaneous; VI, Visceral involvement; LN, Lymph nodes; R, reactive lymph nodes; −, absence of cutaneous, visceral, or lymph node involvement on PET-CT; +, presence of cutaneous, visceral, or lymph node localizations; ++, progression of pre-existent cutaneous, visceral, or lymph node localizations. ∞ 7 SS patients had a CD4/8 ratio < 10 due to prior therapies such as prednisolone, but all fulfilled the WHO-EORTC criteria at the time of diagnosis. * Last known ratio.

**Table 3:** Patients’ expectations at the start of the study period in 6 different domains ranging from “not at all” and “somewhat” to “strongly” and “totally”.

Patient Nr.	Pain	Time Consumption	Mood	Energy	Treatment Effect on Symptoms	Adverse Events
*Patient 1*	Not at all	Somewhat	Not at all	Not at all	Somewhat	Somewhat
*Patient 2*	Not at all	Not at all	Not at all	Not at all	Totally	Not at all
*Patient 3*	Not at all	Strongly	Not at all	Not at all	Somewhat	Somewhat
*Patient 4*	Somewhat	Strongly	Somewhat	Somewhat	Strongly	Somewhat
*Patient 5*	Not at all	Not at all	Not at all	Not at all	Totally	Somewhat
*Patient 6*	Somewhat	Somewhat	Not at all	Somewhat	-	-
*Patient 7*	-	-	-	-	-	-
*Patient 8*	Somewhat	Strongly	Somewhat	Somewhat	Somewhat	Somewhat
*Patient 9*	Not at all	Strongly	Not at all	Somewhat	Totally	Strongly
*Patient 10*	Not at all	Somewhat	Not at all	Somewhat	Totally	Somewhat
*Patient 11*	Somewhat	Strongly	Not at all	Somewhat	Strongly	Somewhat
*Patient 12*	Not at all	Strongly	Not at all	Somewhat	Strongly	Somewhat
*Patient 13*	Somewhat	Strongly	Somewhat	Somewhat	Strongly	Strongly

-, missing data.

**Table 4:** Skindex-29, RAND-12, and itch, pain, and fatigue VAS scores at the start and end of the study period including median scores.

		Skindex-29				Rand-12		VAS		
Patient Nr.		Symptoms	Emotions	Functioning	Total	PCS	MCS	Itch	Pain	Fatigue
*Patient 1*	M1	Mildly (39)	Unaffected (23)	Mildly (23)	Mildly (27)	34	42	3	1	5
	M2	Moderately (46)	Unaffected (23)	Mildly (27)	Mildly (30)	38	36	3	3	5
*Patient 2*	M1	Severely (64)	Severely (53)	Severely (52)	Severely (55)	48	42	8	8	4
	M2	Unaffected (11)	Unaffected (5)	Unaffected (4)	Unaffected (6)	47	47	1	1	1
*Patient 3*	M1	Severely (78)	Unaffected (23)	Severely (54)	Severely (49)	29	34	8	6	5
	M2	Severely (64)	Moderately (38)	Severely (65)	Severely (55)	31	23	7	7	4
*Patient 4*	M1	Severely (61)	Severely (58)	Severely (48)	Severely (54)	35	30	8	7	5
	M2	Severely (78)	Severely (78)	Severely (79)	Severely (78)	32	24	8	7	6
*Patient 5*	M1	Severely (71)	Severely (48)	Severely (52)	Severely (55)	34	30	10	7	8
	M2	Severely (71)	Moderately (38)	Severely (60)	Severely (55)	32	52	8	7	8
*Patient 6*	M1	Severely (61)	Unaffected (15)	Unaffected (19)	Mildly (28)	52	51	7	7	1
	M2	Unaffected (32)	Unaffected (8)	Unaffected (13)	Mildly (16)	57	54	2	2	7
Patient 7 *	M1	Moderately (50)	Severely (40)	Moderately (35)	Moderately (41)	41	30	6	2	6
	M2	-	-	-	-	-	-	-	-	-
*Patient 8*	M1	Moderately (50)	Moderately (38)	Mildly (29)	Moderately (37)	41	24	7	1	7
	M2	Unaffected (36)	Mildly (25)	Severely (44)	Moderately (35)	29	25	2	0	5
*Patient 9*	M1	Severely (93)	Severely (60)	Severely (65)	Severely (70)	34	30	10	9	9
	M2	Unaffected (14)	Moderately (38)	Unaffected (17)	Unaffected (23)	54	44	0	1	6
Patient 10 *	M1	Severely (82)	Severely (70)	Severely (54)	Severely (66)	26	28	9	7	8
	M2	-	-	-	-	-	-	-	-	-
*Patient 11*	M1	Severely (64)	Moderately (38)	Severely (40)	Severely (45)	39	32	10	2	6
	M2	Severely (79)	Severely (50)	Severely (63)	Severely (62)	22	31	9	4	3
*Patient 12*	M1	Severely (68)	Unaffected (15)	Moderately (33)	Moderately (35)	46	48	5	4	5
	M2	Severely (79)	Mildly (28)	Moderately (35)	Moderately (43)	51	51	7	2	5
*Patient 13*	M1	Severely (86)	Severely (40)	Severely (46)	Severely (53)	23	48	9	8	8
	M2	Unaffected (29)	Unaffected (20)	Severely (38)	Mildly (29)	28	38	1	1	8
Total sample (*n* = 13)	M1	64 (55–80)	40 (23–55)	46 (31–53)	49 (36–55)	35 (32–44)	31 (29–51)	8 (7–10)	7 (2–8)	6 (1–8)
	M2	46 (29–79)	28 (20–38)	38 (17–63)	35 (23–55)	32 (30–45)	38 (25–51)	3 (1–8)	2 (1–7)	6 (5–8)

Numbers for the total group are presented as median (IQR). PSC, Physical Component Score; MCS, Mental Component Score; VAS, Visual Analogue Scale; M1, baseline measurement; M2, measurement after follow-up. - missing data. * Patient died during the follow-up.

**Table 5:** Treatment satisfaction as measured with the CSQ-8.

	Treatment Satisfaction								
Patient Nr.	Quality of Treatment	Type of Treatment	Met Needs	Recommend to a Friend	Amount of Help	Deal with Problems	Overall Satisfaction	Come Back	Total (25–100)
*Patient 1*	Good	Yes, generally	Most of my needs have been met	Yes, definitely	Very satisfied	Yes, helped a great deal	Very satisfied	Yes, definitely	91
*Patient 2*	Excellent	Yes, definitely	Almost all of my needs have been met	Yes, definitely	Very satisfied	Yes, helped a great deal	Very satisfied	Yes, definitely	100
*Patient 3*	Good	Yes, generally	Most of my needs have been met	Yes, generally	Mostly satisfied	Yes, somewhat	Mostly satisfied	Yes, I think so	75
*Patient 4*	Fair	Yes, generally	Only a few of my needs have been met	Yes, generally	Quite, dissatisfied	No, not really	Mostly satisfied	Yes, I think so	59
*Patient 5*	Excellent	No, definitely not	Only a few of my needs have been met	Yes, definitely	Indifferent or mildly dissatisfied	No, not really	Very satisfied	Yes, definitely	72
*Patient 6*	Excellent	Yes, definitely	Most of my needs have been met	Yes, generally	Mostly satisfied	Yes, helped a great deal	Very satisfied	Yes, definitely	91
Patient 7 *	-	-	-	-	-	-	-	-	-
*Patient 8*	Good	Yes, generally	Most of my needs have been met	Yes, generally	Mostly satisfied	Yes, helped a great deal	Very satisfied	Yes, I think so	81
*Patient 9*	Excellent	Yes, definitely	Almost all of my needs have been met	Yes, definitely	Very satisfied	Yes, helped a great deal	Very satisfied	Yes, definitely	100
Patient 10 *	-	-	-	-	-	-	-	-	-
*Patient 11*	Fair	No, definitely not	None of my needs have been met	No, not really	Indifferent or mildly dissatisfied	No, not really	Indifferent or mildly dissatisfied	No, definitely not	41
*Patient 12*	Fair	No, not really	Only a few of my needs have been met	No, not really	Quite, dissatisfied	No, not really	Indifferent or mildly dissatisfied	Yes, I think so	50
*Patient 13*	Excellent	Yes, definitely	Almost all of my needs have been met	Yes, definitely	Mostly satisfied	Yes, helped a great deal	Very satisfied	Yes, definitely	97
Total score	-	-	-	-	-	-	-	-	81 (59–79)

Numbers for the total score are presented as median (IQR). - missing data. * Patient died during the follow-up.

## Data Availability

Data will be available upon reasonable request via the corresponding authors.
